# Avian Influenza: Mixed Infections and Missing Viruses

**DOI:** 10.3390/v5081964

**Published:** 2013-08-05

**Authors:** LeAnn L. Lindsay, Terra R. Kelly, Magdalena Plancarte, Seth Schobel, Xudong Lin, Vivien G. Dugan, David E. Wentworth, Walter M. Boyce

**Affiliations:** 1 Department of Pathology, Microbiology and Immunology, University of California, One Shields Avenue, Davis, CA 95616, USA; E-Mails: lllindsay@ucdavis.edu (L.L.L.); mplancarte@ucdavis.edu (M.P.); 2 One Health Institute, University of California, 1089 Veterinary Medicine Drive, Davis, CA 95616, USA; E-Mail: trkelly@ucdavis.edu; 3 J. Craig Venter Institute, Rockville, MD 20850, USA; E-Mails: sschobel@jcvi.org (S.S.); xulin@jcvi.org (X.L.); vgdugan@hotmail.com (V.G.D.); dwentwor@jcvi.org (D.E.W.)

**Keywords:** avian influenza, surveillance, hemagglutinin, virus isolation, embryonated chicken egg, sequencing, genome

## Abstract

A high prevalence and diversity of avian influenza (AI) viruses were detected in a population of wild mallards sampled during summer 2011 in California, providing an opportunity to compare results obtained before and after virus culture. We tested cloacal swab samples prior to culture by matrix real-time PCR, and by amplifying and sequencing a 640bp portion of the hemagglutinin (HA) gene. Each sample was also inoculated into embryonated chicken eggs, and full genome sequences were determined for cultured viruses. While low matrix Ct values were a good predictor of virus isolation from eggs, samples with high or undetectable Ct values also yielded isolates. Furthermore, a single passage in eggs altered the occurrence and detection of viral strains, and mixed infections (different HA subtypes) were detected less frequently after culture. There is no gold standard or perfect reference comparison for surveillance of unknown viruses, and true negatives are difficult to distinguish from false negatives. This study showed that sequencing samples prior to culture increases the detection of mixed infections and enhances the identification of viral strains and sequences that may have changed or even disappeared during culture.

## 1. Introduction

Influenza A surveillance in wild birds is crucial to understanding the origin, evolution and transmission of viruses that pose a potential health risk for humans, domesticated animals, and wildlife [1,2,3,4]. Ideally, field and laboratory efforts should yield results that accurately reflect nature—screening tests should have 100% sensitivity and specificity, and virus isolation and sequencing should not alter or bias results. However, this is rarely the case, and many studies have shown that researchers must carefully consider the limitations and biases associated with different sampling strategies and diagnostic tests [5,6,7,8,9,10].

On August 12 of 2011, we sampled 79 mallards (*Anas platyrhynchos*) at a single location in northern California that had a high prevalence (>50% positive by matrix gene real-time PCR) and diversity of hemagglutinin (HA) subtypes (H3, H4, H5, H6, H11, and H12) of avian influenza (AI) viruses. These samples provided a resource to evaluate and compare methods commonly used for AI surveillance in wild birds, and to examine how results differed before and after virus culture. We were particularly interested in exploring how culture affected our ability to detect mixed infections and the full range of viral diversity present in the ducks. Duplicate cloacal samples from each bird were screened for virus nucleic acid by matrix real-time (RT) PCR, and these pre-culture samples were subtyped by conventional PCR/sequencing of a 640 bp section of the HA gene. Each sample was inoculated and cultured in embryonated chicken eggs (ECE) to detect live virus, and allantoic fluid (ALF) was tested by matrix real time PCR, HA-subtyped by conventional PCR, and full genomes sequenced by a multisegment-RTPCR and next generation sequencing (NGS). 

## 2. Results

### 2.1. AI Matrix Real-Time PCR of VTM and ALF Samples

Fifty-one percent (81/158) of the duplicate swab samples (labeled P and V) had AI matrix real-time PCR cycle threshold (Ct) values <45 (the limit of detection), and 58% of the birds (46/79) were positive at this cutoff for either their P or V sample (Table 1). Similar values were obtained when repeat testing of the original samples was performed one year later, indicating test results were highly reproducible (*p* < 0.05); only average Ct values are reported. When Ct values for P and V samples (n = 79 each) were compared, there was no significant difference (*p* > 0.05) in the frequency of matrix-positive results, and kappa statistics (>0.80) indicated very good, but not perfect, agreement (Table 1).

**Figure 1:**
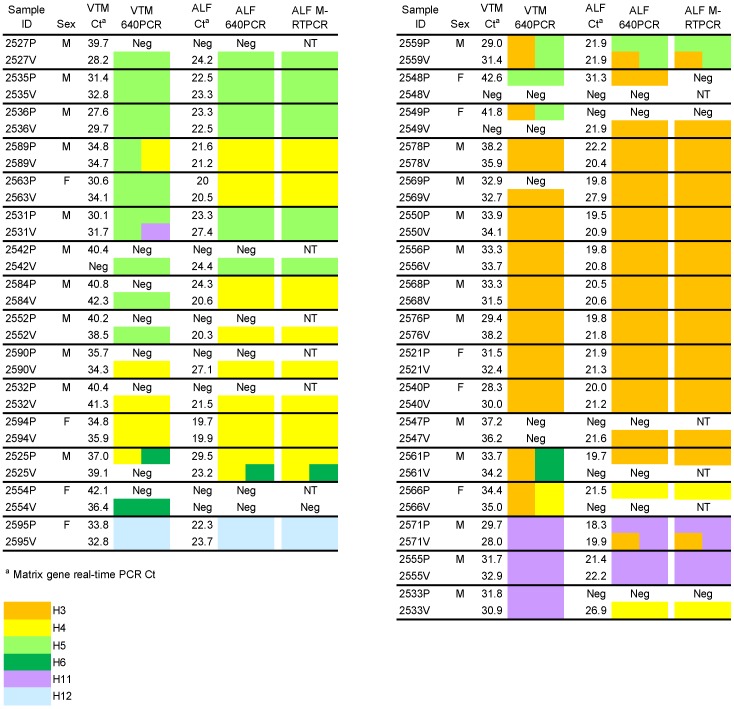
Matrix real-time PCR and HA subtyping results for paired samples (P, V) from 32 birds before and after inoculation in ECE. HA subtypes in the original VTM samples were determined by 640PCR/Sanger sequencing, while subtypes in allantoic fluid (ALF) were determined by both 640PCR and M-RTPCR/NGS. NT = not tested. Neg = matrix Ct >45 or HA subtype not detected.

Thirty-two percent (51/158) of the samples inoculated into ECE yielded matrix-positive ALF samples, representing 39% of the birds (31/79) (Table 1). As shown in Figure 1, the Ct values for positive ALF samples (Ct 18-31) were much lower than positive VTM samples (Ct 27-43), almost certainly as infection and replication produced larger amounts of viral RNA. As expected, there was a strong relationship between Ct values of matrix-positive VTM samples and the likelihood of egg inoculation yielding matrix-positive ALF. Overall, 60% (49/81) of the matrix PCR-positive VTM samples grew in eggs; two matrix-negative samples also grew in ECE for 51/81 samples total (Figure 1 and Table 1).

**Table 1:** Detection of influenza A matrix and hemagglutinin (HA) genes before (VTM samples) and after (ALF samples) inoculation in ECE. Paired cloacal swab samples (P, V) were tested from 79 mallards.

Sample Unit	Pre-inoculation (VTM)			Post-inoculation (ALF)		
	Matrix Ct < 45	Matrix Ct < 35	HA positive (640PCR)	Matrix Ct < 45	640PCR HA positive	M-RTPCR HA positive
P swab	56% (44/79)	25% (20/79)	29% (23/79)	29% (23/79)	29% (23/79)	28% (22/79)
V swab	47% (37/79)	24% (19/79)	35% (28/79)	35% (28/79)	35% (28/79)	35% (28/79)
P and V swabs	51% (81/158)	25% (39/158)	32% (51/158)	32% (51/158)	32% (51/158)	32% (51/158)
Birds	58% (46/79)	28% (22/79)	39% (31/79)	39% (31/79)	39% (31/79)	38% (30/79)

### 2.2. HA Subtyping

Thirty-two percent (51/158) of the VTM and ALF samples were subtyped by either 640PCR or M‑RTPCR. Figure 1 shows the HA subtyping results for the 32 birds that yielded an HA subtype by 640PCR and/or M-RTPCR in at least one of the duplicate VTM samples or corresponding ALF samples. Six HA subtypes (H3, H4, H5, H6, H11, and H12) were identified, with H3, H4, and H5 being the most common. All H5 sample sequences were low pathogenicity AI. Mixed infections were detected in seven of the bird VTM samples, while only three samples showed mixed infections in the ALF. There were no significant subtype differences between male and female birds. The agreement between ALF subtypes determined by 640PCR and M-RTPCR was nearly perfect; only two cases were noted where there was not a perfect match (Figure 1 and Table 1). However, the ALF subtypes differed from the VTM subtype for 15 of the 46 samples where both the VTM and ALF samples were subtyped, corresponding to conflicting results for 12 of 32 birds (kappa = 0.5 (0.25–0.75)). 

### 2.3. M-RTPCR Virus Segment Amplification

**Figure 2:**
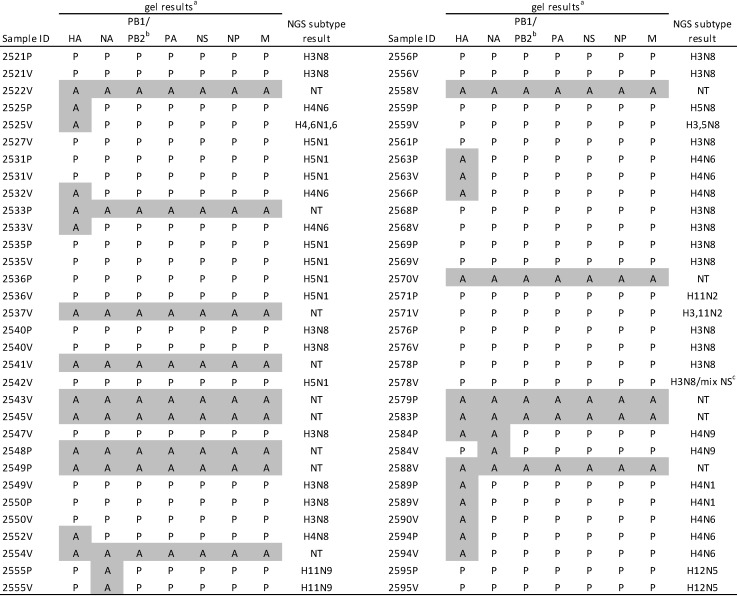
Multi-segment RT-PCR results for allantoic fluid (ALF) samples that were positive by matrix real-time PCR. Full genome sequences were determined by next generation sequencing (NGS) for samples even if they lacked detectable bands. ^a^ Agarose gel profiles were examined for the presence/absence of individual segment bands. If all segment bands were absent on the gel, the sample was not put through NGS. Absent bands are highlighted. P = band present, A = band absent, NT = not tested by NGS and reported as negative in Figure 1. ^b^ The PB1 and PB2 segments are the same size and therefore cannot be resolved on agarose gels. ^c^ Sample 2578V was found to have two NS sequences so was designated “H3N8 mix.”

The M-RTPCR amplicons were analyzed by agarose gel electrophoresis prior to NGS to assess whether all eight segments were present. Figure 2 shows the banding patterns for the amplified products, scoring samples for the presence/absence of specific segment bands. In every case where bands were observed but not all segments were present, the segments below the limit of detection were either HA or NA or both; all of the internal segment bands were present. These samples still yielded full genome sequences upon subsequent NGS. Interestingly, all of the samples where the HA band was missing but an HA sequence was still obtained (n = 14) were H4, with only one H4 sample showing a band on the gel. All of the NA subtyped samples showing missing NA bands (n = 4) were N9, and this represented all of the N9 samples.

### 2.4. Sequence Analysis of Duplicate Samples from Birds

Full genome sequences from virus isolates were obtained from both the P and V swabs for 20 birds, allowing comparison of the percent nucleotide identities for the eight genome segments (Table 2). Of the total of 160 segment pairs, 46 (29%) showed sequence variability, ranging from 91.63%–99.96% nucleotide identity, with 23 showing <99.90% identity. The HA and NA segments showed the least amount of variability, while the NP and NS segments showed the most. One sample, 2578V, showed a mixed NS sequence, therefore it was designated “H3N8 mix.” As expected, birds exhibiting mixed infections had lower percent identity values between sample segments due to differing virus sources. However, there was less variation than anticipated. In many cases the segment sequences were identical even though the presence of more than one virus was indicated. 

**Table 2:** Comparison of nucleotide identities for viruses isolated from different swabs from the same bird. Twenty birds yielded viruses from both their P and V swabs when inoculated into ECE.

Bird ID	NGS Subtype	PB2^a^ % identity	PB1 % identity	PA % identity	HA % identity	NP % identity	NA % identity	M % identity	NS % identity
2521P	H3N8	100	100	100	100	100	100	100	100
2521V	H3N8								
2525P	H4N6	99.96	100	100	100 (H4)	100 (P,Va)^b^	100	100	100
2525V	H4,6N1,6					92.22 (P,Vb)			
2531P	H5N1	98.14	100	99.77	99.94	91.76	100	98.8	93.43
2531V	H5N1								
2535P	H5N1	100	100	99.91	100	100	100	100	100
2535V	H5N1								
2536P	H5N1	100	99.91	100	99.94	99.93	100	100	100
2536V	H5N1								
2540P	H3N8	98.92	99.31	99.82	99.94	99.93	100	100	100
2540V	H3N8								
2550P	H3N8	100	100	100	100	100	100	100	100
2550V	H3N8								
2555P	H11N9	100	100	100	99.94	100	100	100	99.88
2555V	H11N9								
2556P	H3N8	100	99.91	100	100	100	100	100	100
2556V	H3N8								
2559P	H5N8	99.05	96.79	96.51	99.94 (H5)	91.63 (P,Va)	100	97.6	93.53
2559V	H3,5N8					99.93 (P,Vb)			
2563P	H4N6	100	99.91	100	99.94	91.9	99.72	98	100
2563V	H4N6								
2568P	H3N8	100	99.96	100	100	100	100	100	100
2568V	H3N8								
2569P	H3N8	100	100	100	100	100	100	100	100
2569V	H3N8								
2571P	H11N2	100	100	100	99.88 (H11)	100	100	100	100
2571V	H3,11N2								
2576P	H3N8	100	100	100	100	100	99.93	100	100
2576V	H3N8								
2578P	H3N8	100	97	99.95	100	98.69	99.93	100	100 (P,Va)
2578V	H3N8 mix								93.87 (P,Vb)
2584P	H4N9	99.96	100	100	99.71	100	100	100	100
2584V	H4N9								
2589P	H4N1	99.91	100	100	99.94	100	100	99.9	100
2589V	H4N1								
2594P	H4N6	100	100	100	100	100	100	99.9	100
2594V	H4N6								
2595P	H12N5	100	100	100	100	100	100	100	100
2595V	H12N5								

^a^ When both of the swab ALF samples (P and V) from a given bird yielded sequences for each of the eight viral genome segments by M-RTPCR/NGS, percent nucleotide identity was calculated. Highlighted samples were from samples that showed evidence of mixed infections within the VTM or ALF or both in the P and/or V samples (see Figure 1 and text for bird 2578). ^b^ When two sequences were found within a sample they were labeled “a” and “b.”

## 3. Discussion

The matrix gene real-time PCR is widely used to estimate the prevalence of AI in wild bird populations, and the assay is sometimes used to screen samples for subsequent virus isolation [5,8,10,11,12]. Matrix Ct values are inversely related to the amount of viral RNA present in a sample-samples with low CT values have more viral RNA while samples with less RNA must undergo additional cycles to reach the threshold response. Although the maximum limit of detection for the matrix PCR assay is 45, many studies have shown that virus isolation success is much higher for samples having lower (<35) Ct counts [7,9,13]. In the present study, viruses were isolated from 39% of birds indicating that these birds were shedding infectious virus (Figure 1 and Table 1). Virus was isolated from a single swab for 11 of 31 birds, most often corresponding to VTM samples that had high matrix Ct values (=low amount of RNA). Eight VTM samples with Ct values >35 grew in eggs, including two with values above detectable limits (>45), while three samples with Ct values <35 did not grow. Thus, as shown in previous studies [5,9,10,11,12,13] the VTM matrix Ct value is a good, but imperfect predictor for virus growth in ECE, and several isolates would have been missed if a low matrix Ct value were used as a prerequisite for inoculation into eggs. 

We amplified and sequenced a portion of the HA gene to provide an independent assessment (other than matrix assay) of AI infection in samples prior to egg inoculation. As with the matrix assay, the 640PCR test only detects viral RNA and does not indicate whether infectious virus is present. Thirty‑nine percent of birds (31/79) were HA positive on one or both of their VTM samples (Table 1), and six HA subtypes were detected (H3, H4, H5, H6, H11, H12). Mixed infections (two HA subtypes) were detected in 11 swab samples from a total of seven birds. All of the swab samples with matrix Ct values <35 were able to be HA subtyped, likely because these samples had the most viral RNA template for both the matrix and HA genes. However, 17 additional HA-subtyped samples were obtained by sequencing samples with matrix Ct values >35, demonstrating that samples with high matrix Ct values can provide important genotype information prior to virus culture and amplification (Figure 1 and Table 1). 

The detection of HA sequences in VTM samples was a good predictor of virus growth in ECE, and >90% of HA positive samples (46/51) yielded virus isolates. Conversely, growth in eggs was a good indicator that HA sequences could be obtained from the original VTM sample. Of the 51 VTM samples that yielded virus isolates, only five could not be HA subtyped prior to inoculation in eggs. Although the overall frequency of HA positive results did not differ between paired swab samples, 35% (11/31) of the HA positive birds had only a single positive swab pre-inoculation. As with the matrix gene PCR test, combining the results of the two samples from each bird increased the estimated prevalence of AI within the population, and it provided a more accurate view of which birds were shedding infectious virus. These results are consistent with other studies that showed a combination of methods improved the accuracy of detection [6,10].

It is widely recognized that virus culture in ECE is a necessary, but imperfect method for isolating influenza viruses infecting wild birds [5,7,8,9,10,12], and comparison of HA subtypes obtained before and after egg inoculation showed that egg culture altered the occurrence and detection of viral strains. Although the same six HA subtypes were detected in birds before and after egg inoculation, the detection of H3 and H4 subtypes together increased in frequency from 58% to 77% post-inoculation, while H5 detections decreased by 50%. Mixed infections were also detected less frequently, as has been suggested by other direct studies of VTM samples [14], occurring in seven birds pre-inoculation but in only three birds post-inoculation. These changes likely reflect low amounts or a lack of viable virus in the original sample, variable fitness of different strains to grow in ECE, and/or virus competition in mixed infections [7,10,15,16,17]. Most importantly, these results show that culture in eggs provides a biased view of viruses present in original or primary samples. The ideal surveillance assay would yield full genome sequences from original samples without culture. Based on the results of the present study, we now perform both 640PCR and M-RTPCR assays to obtain partial and even full genome sequences from primary VTM samples. However, the low amount of RNA present in many samples poses a major obstacle, and more sensitive methods are needed. 

The multi-segment M-RTPCR and the 640PCR gave essentially identical results for subtyping virus isolates in ALF. Although M-RTPCR amplification of a subset of HA and NA segments was inefficient in some cases, subsequent NGS resulted in complete genome sequences. This indicated that all eight RNA segments were present in the ALF samples as expected, but that the HA/NA segments were not efficiently amplified during the M-RTPCR reaction. Interestingly, the poor amplification was restricted to H4 and N9 in our sample set, perhaps because RNA secondary structure inhibited efficient transcription, or the PCR conditions were not optimal for these subtypes. Decreased efficiency of H4 amplification compared to other HA subtypes has also been reported in a study using a different PCR protocol that also used primers based on the conserved 5' and 3' ends of the viral segments [18]. 

Virus selection and possibly mutation in ECE was observed by comparing the sequences obtained from duplicate AI isolates (two swab samples from the same bird). Nucleotide differences were observed in 29% of the paired segments (n = 20 birds; 160 pairwise comparisons), with identities as low as 91.63%. Variability between paired samples with high identity (>99.9%) may have been caused by the high error rate (≤0.1%) of virus RNA polymerase [1], as well as PCR and DNA sequencing polymerase errors [19]. Paired samples that showed <99.9% identity among one or more segments (14% of total, n = 23 segment pairs) were from birds (8/20) with mixed infections, *i.e.*, two HA subtypes were detected in the ALF sample, or, more commonly, there were differing HA subtypes detected in the VTM sample *versus* the corresponding ALF. However, not all segments in these mixed samples showed variability, likely because the different virus strains shared common segment sequences as observed previously [3]. Another possibility is that during growth in the ECE, certain virus segments from the mix were retained and others “discarded” during reassortment and virus packaging. Non-random reassortment of segments in mixed infections has been observed in other studies, and it has been postulated that strain-specific signals in certain genomic segments result in selective packaging in progeny virus particles [17,20,21]. 

This study showed that mixed infections and naturally occurring viruses can be overlooked or altered by laboratory methods, and that analysis of primary samples prior to culture provides a more accurate view of viruses occurring *in vivo*. However, despite the use of increasingly sophisticated tools, our attempts to identify and characterize viral diversity will continue to be challenged by the dynamic, quasispecies nature of influenza viruses [22,23]. The diversity of subtypes and mixed infections we detected prior to culture, and the nucleotide differences we observed in sequences from paired swabs from the same bird, hint at the underlying complexity of infection likely occurring at the cellular level. However, consensus sequences oversimplify the genetic diversity and complexity of influenza virus infection *in vivo*, and they fail to fully convey a given isolate’s potential for cross-species transmission and pathogenicity [24]. Deep sequencing efforts that focus on characterizing the full diversity and interactions among virus subpopulations at the molecular and cellular levels will be essential going forward, particularly if they are applied to primary samples not altered by sample handling and laboratory methods [25].

## 4. Conclusions

The true infection status of the 79 birds in this study was unknown, as is the case for all wild bird surveillance. There is no gold standard or perfect reference comparison for surveillance of unknown viruses; therefore, true negatives and false negatives cannot be reliably distinguished, and each additional sample and diagnostic test can provide additional information. Virus culture, with all its limitations, continues to be necessary for obtaining sufficient amounts of RNA and live virus for genotypic and phenotypic characterization. However, careful thought should be given to making sure that inferences drawn from the study of cultured viruses apply to the viruses that actually occur in nature. Improved methods for analyzing primary samples and sequencing viruses without previous culture will help ensure that naturally occurring variants are detected. 

## 5. Experimental Section

### 5.1. Samples

Wild mallard ducks (n = 79) were captured and sampled on 12 August 2011 in the Suisun Marsh near San Francisco, CA (38.1307°N, 121.9814°W). Influenza virus infection and shedding occurs seasonally among mallards at this site [26], and most of the captured ducks (74/79) were hatch-year birds <6 months of age (51 males, 28 females). Cloacal swabs were collected in duplicate (79 birds = 158 swab samples) and placed in separate vials containing 2 mL of ice-cold virus transport medium (VTM: Medium 199 with Earle’s salts, L-glutamine, and sodium bicarbonate, plus 2 mU/L penicillin G, 200 mg/L streptomycin, 2 mU/L polymyxin B, 250 mg/L gentamicin, 0.5 mU/L nystatin, 60 mg/L ofloxacin, 200 mg/L sulfamethoxazole, and 0.5% bovine serum albumin V) [27]. The samples were transported on ice to the laboratory where they were stored at −80 °C. The duplicate samples were designated “P” (for PCR) and “V” (for virus isolation), and were collected in random order. In the laboratory, P samples were thawed to withdraw aliquots for RNA extraction and PCR, and then refrozen. The remaining P samples were later inoculated into ECE after a total of two or three freeze-thaw cycles. The V samples were inoculated into ECE after a single freeze-thaw cycle, refrozen, and then later thawed again for RNA extraction and PCR. 

### 5.2. RNA Isolation and Reverse Transcription

The VTM samples were thawed on ice, vortexed briefly, and 300 µL was centrifuged in a microcentrifuge at top speed for 3 min. All but ~50 µL of the supernatant was removed, and the pellet was resuspended with the addition of 50 µL of lysis buffer (without carrier RNA) from a MagMAX-96 AI/ND Viral RNA Isolation Kit (Ambion Inc. Austin, TX, USA). A Kingfisher nucleic acid extraction system (Thermo Scientific) was used to extract RNA according to the MagMAX kit instructions. The RNA samples were stored at −80 °C until use. Complementary DNA was generated from 4 µL of RNA using a MMLV reverse transcriptase kit (Invitrogen, Carlsbad, CA, USA) following the manufacturer’s protocol and using random primers. 

### 5.3. Influenza A PCR

Influenza A positive VTM and ALF samples were identified by real-time PCR to amplify the matrix gene based on a protocol by Spackman *et al.* [28] and modified by Runstadler *et al.* [11]. A TaqMan Universal PCR Master Mix No AmpErase UNG Kit (Applied Biosystems, Branchburg, NJ, USA) was used with 3 µL cDNA in a 25 µL reaction volume containing 800 nM primers (forward primer, 5' ARATGAGTCTTCTRACCGAGGTCG 3', reverse primer, 5' TGAAAAGACATCYTCAAGYYTCTG 3') and 200 nM probe ([6-FAM]TCAGGCCCCCTCAAAGCCGA[TAMRA-6-FAM]). The PCR program consisted of 95 °C for 10 min followed by 45 cycles of 95 °C for 15 s and 65 °C for 1 min. Results from real-time PCR are reported in threshold cycle (Ct) values, which correspond to the number of PCR cycles required to detect nucleic acid (lower Ct levels indicate greater concentration of virus RNA in the sample). Any sample with a Ct value ≥45, *i.e.*, exceeding the maximum number of cycles specified by the real-time PCR program, was considered negative (beyond detectable limits). An Applied Biosystems 7500 Fast instrument was used. All samples were tested twice, each time starting with the extracted RNA.

### 5.4. Virus Isolation

All VTM samples (P and V) were inoculated into 11-day-old ECE (Charles River, North Franklin, CT, USA) following standard methods [27]. A total of 4 eggs were used to isolate viruses from each bird, since each of the paired samples from a single bird was inoculated into 2 eggs. The VTM was thawed, vortexed briefly, and 200 µL removed and mixed with 100 µL of an antibiotic/anti-fungal solution containing streptomycin sulphate, penicillin G, polymyxin B, nystatin, ofloxacin, gentamycin and sulfamethoxazole [29]. The mixture was injected into the allantoic cavities of two eggs followed by incubation at 37.8 °C for three days or until embryo death as detected by daily candling. The ALF from each pair of eggs was harvested, pooled, and 50 µL was immediately processed for RNA extraction, cDNA generation, and PCR testing as described above. Remaining ALF was frozen at −80 °C. Second passages in eggs were not attempted so as to avoid selection for egg-adapted mutants. 

### 5.5. HA Subtyping

HA subtyping was performed on cDNA from all VTM and ALF samples using a modified protocol based on Phipps *et al.* [30] to generate a 640 bp product (640PCR). The forward primer 5' GGRATGRTHGAYGGNTGGTAYGG 3' was a modification of the Phipps *et al.* [30] primer to include HA sequences often found in California. The HARK reverse primer 5' ATATGGCGCCGTATTAGTAGAAACAAGGGTGTTTT 3' was from Bragstad *et al.* [31]. The 25 µL PCR reaction used 0.08 U Amplitaq Gold polymerase and 10× buffer without MgCl_2_ (Invitrogen), 1.5 mM MgCl_2_, 0.2 mM dNTP mix, and 0.6 µM of each primer. The PCR program consisted of 10 min at 95 °C, followed by 45 cycles of 94 °C for 1 min, 58 °C for 1 min, and 72 °C for 1 min, with a final extension of 72 °C for 7 min. Following electrophoresis on a 1.5% agarose gel, the 640 bp band was excised and purified using a QIAquick gel extraction kit (Qiagen, Valencia, CA, USA) with an elution volume of 30 µL. If a 640 bp product was not obtained using an annealing temperature of 58 °C, the temperature was reduced to 50 °C. Direct Sanger sequencing of amplicons was performed (College of Biological Sciences ^UC^DNA Davis Sequencing Facility, University of California, Davis, CA, USA) using the HARK reverse primer. Sequences were subtyped by performing a BLAST search at [32] to find the closest possible match from available HA sequences in GenBank. When mixed sample sequences were obtained, the chromatograms were inspected by eye and in several cases one sequence dominated, thus the two sequences could be distinguished. If mixed infections were indistinguishable, the PCR products were cloned using a pGEM-T Easy vector system (Promega, Madison, WI, USA) using the manufacturer’s instructions. Multiple colonies (6–10) were processed for sequencing. Subtypes were also determined by the J. Craig Venter Institute (JCVI) using a multi-segment reverse transcription-PCR (M-RTPCR) to simultaneously amplify all eight influenza A genome segments in a single reaction [33,34]. Full genome sequencing was then performed by high‑throughput NGS [19]. The Genbank accession numbers for these segment nucleotide sequences are CY133917 through CY134294.

### 5.6. Statistical Analyses

McNemar’s test was used to evaluate differences in results between P and V swabs (real-time PCR, 640PCR, M-RTPCR), to compare the frequencies of matrix-positive VTM and ALF samples, and to compare frequencies of HA positive results by 640PCR and M-RTPCR. Differences in the distribution of HA subtypes by sex was assessed using Fisher’s Exact test. Agreement in results between HA subtyping protocols and between the P and V swabs was evaluated using Cohen’s Kappa statistic with the following guidelines for index interpretation: poor agreement (<0.00), slight agreement (0.00–0.20), fair agreement (0.21–0.40), moderate agreement (0.41–0.60), good agreement (0.61–0.80) and very good agreement (0.81–1.00) [35]. All analyses were performed using R [36]. A *p*-value < 0.05 was considered statistically significant. 
